# Day‐To‐Day Endurance Training Periodization of World‐Class Cross‐Country Skiers

**DOI:** 10.1002/ejsc.12322

**Published:** 2025-05-21

**Authors:** Jacob Walther, Jan Kocbach, Øyvind Sandbakk

**Affiliations:** ^1^ Department of Sports Medicine and Exercise Therapy Institute of Human Movement Science and Health Chemnitz University of Technology Chemnitz Germany; ^2^ Department of Neuromedicine and Movement Science Centre for Elite Sport Research Norwegian University of Science and Technology Trondheim Norway; ^3^ School of Sport Science UiT the Artic University of Norway Tromsø Norway

## Abstract

This study aimed to investigate the day‐to‐day endurance training periodization of male and female world‐class cross‐country skiers. Self‐reported session‐by‐session training data from 17 (7 female) world‐class cross‐country skiers were retrospectively analyzed. The data were separated into general preparation (GP) and competition periods (CP), during which all skiers achieved world‐class performance. Daily training volume was categorized as very low (0%–50% of mean daily volume), low (50%–100%), moderate (100%–150%), and high (≥ 150%). Training load (Training Impulse, TRIMP) was calculated as the product of duration and an intensity factor of 1, 2, or 3 for low‐, moderate‐, and high‐intensity training, respectively. Training volume was distributed as 18%/22%/32%/28% across very low/low/moderate/high volume days during GP, whereas the corresponding distribution during CP was 23%/35%/32%/10%. Shifts in daily TRIMP from GP to CP mirrored changes in training volume. During GP, the acute‐to‐chronic work ratio (ACWR) was 0.75–1.25 on 69% of the days, with 13% of the days showing lower ratios and 18% higher. During CP, the corresponding values were 69%/19%/12%. Intensive sessions occurred every 3.6 ± 0.3 days during GP compared to every 3.0 ± 0.3 days during CP. Training volumes on the day before intensive sessions were 127 ± 13% (GP) and 99 ± 7% (CP) of the daily mean, whereas the corresponding values for the days after were 116 ± 8% and 87 ± 9%. These data provide new insights into day‐to‐day periodization in world‐class cross‐country skiers, highlighting the sequential application of training loads by alternating training days of high volume, elevated intensity every 3–4 days, or reduced training load or rest every 8–9 days, while only 5% combining volume and intensity.

**Summary**

- The day‐to‐day periodization of world‐class cross‐country skiers was characterized by a system where the applied training sequences are distributed in an altering manner with days of high volume, days with elevated intensity, and only 5% of days combining elevated volume and intensity.
- A balanced organization of daily loads including days of reduced loads, full rest days every 8–9 days, and a relatively steady acute to chronic load evolvement (∼15% of the days having an ACWR ≥ 1.25) was applied to ensure consistency.
- Intensive sessions were separated by 3–4 days, although time gaps between sessions of the same intensity were generally larger. During the competition period, intensive sessions were concentrated around competitions with frequent back‐to‐back intensive days.

## 1 Introduction

During the past decades, numerous studies have described the training characteristics of elite cross‐country (XC) skiers, quantifying annual training volume, training forms, intensity distribution, and exercise modalities (Tønnessen et al. 2014; Solli et al. 2017; Walther et al. 2023; Ø. Sandbakk and Holmberg 2014, 2017). During successful seasons, world‐class XC skiers of both sexes are reported to train 750–1000 h annually, of which 90% is dedicated to endurance training and the remaining 10% to strength and speed training (Walther et al. 2023; Ø. Sandbakk and Holmberg 2014, 2017). These training hours are typically distributed across approximately 520 sessions and 310 training days per year (Solli et al. 2017; Walther et al. 2023). Consequently, world‐class XC skiers have about 50 rest days and 200 double‐session days (i.e., days with two sessions) annually (Walther et al. 2024). In terms of endurance training, ∼400 sessions are devoted exclusively to low‐intensity training (LIT) (not including warm‐ups), complemented by ∼80 intensive sessions (moderate‐intensity training [MIT] and high‐intensity training [HIT]) and ∼30 races. This results in an annual training intensity distribution (TID) of approximately 90%, 5%, and 5% of endurance time allocated to LIT, MIT, and HIT, respectively (Walther et al. 2024). This training is commonly organized using a traditional periodization model, where the general preparation period (GP) is characterized by higher volumes of LIT and MIT, whereas training volume decreases and HIT increases as the competition period (CP) approaches (Solli et al. 2017; Walther et al. 2023).

Although descriptions of macro‐ (year(s)) and meso‐ (month‐long) cycles of training load factors provide a valuable starting point, coaches and athletes strive to achieve a purposeful arrangement and sequencing of different training units within these cycles. This process is more complex than the broader periodization across macro‐ and mesocycles (Tønnessen et al. 2014; Solli et al. 2017; Walther et al. 2023). In this context, previous studies have explored the typical session designs employed by world‐class XC skiers, emphasizing LIT sessions lasting 90–150 min and MIT sessions with 40–60 min of intensive work as the most frequently applied designs (Solli et al. 2017; Walther et al. 2024; Tønnessen et al. 2024). However, no evidence currently bridges the gap between session designs and annual training characteristics by investigating the arrangement and sequencing of training in smaller units, such as weeks and days. The proximities between training sessions, combined with phases of elevated volume or intensity and recovery phases, can lead to varying levels of physiological strain and, consequently, different adaptations (Andrade‐Souza et al. 2020; Ghiarone et al. 2019).

Altogether, understanding how world‐class XC skiers distribute their daily training load and how training at different intensities is organized over shorter periods would provide complementary insights. Organizational changes across the training year, from GP to CP, have not yet been systematically examined. Therefore, the aim of this study was to retrospectively investigate the day‐to‐day endurance training periodization strategies of male and female world‐class XC skiers during a successful season.

## 2 Methods

### 2.1 Participants

Seventeen senior world‐class XC skiers (7 women) volunteered to participate in this study. Inclusion criteria were as follows: (1) medalist in either world championships or Olympic Games, or repeated podium finishes in World Cup races and (2) detailed training log data from their most successful senior season. The included skiers were born between 1972 and 1998 (age during the analyzed season: 28.4 ± 2.7 years) and had collectively earned 38 Olympic medals, 69 senior world championship titles, 291 World Cup victories, and 23 junior world championship titles. All participants provided written informed consent to participate in this study. The regional committee for medical and health research ethics waived the requirement for ethical approval for studies of this type. The ethics of the project adhered to institutional requirements at the Department of Neuromedicine and Movement Science, Norwegian University of Science and Technology, Norway. Approval for data security and handling was obtained from the Norwegian Center for Research Data (reference no. 419807).

### 2.2 Study Design

A retrospective study design was used to analyze self‐reported endurance training characteristics during the most successful senior seasons. The most successful senior season for each athlete was selected holistically based on predefined performance parameters. These parameters were assessed in the following order: (1) number of individual medals in world championships and Olympic Games, (2) number of World Cup podium finishes, (3) number of team medals in world championships and Olympic Games, and (4) calculated peak age.

### 2.3 Training Data

All participants reported their training session by session, including information on duration, training form, intensity, and exercise mode, using a diary designed by the Norwegian Top Sport Center (Olympiatoppen). Before the introduction of the web‐based diary, participants used nonweb‐based versions (e.g., Microsoft Excel) or written diaries designed by the Norwegian Ski Federation. The distributions of training forms, intensities, and modes were analyzed as previously described (Walther et al. 2023).

For intensity quantification, a three‐zone scale (LIT, MIT, and HIT; with intensities being separated at the first and second lactate threshold) and the modified session goal approach were employed, as previously described (Walther et al. 2023). For analyses of intensive sessions, the session goal approach was used, meaning sessions were categorized as MIT or HIT if the core part of the session consisted of this intensity. Since athletes may use multiple intensities within the same session, intensive sessions were categorized as MIXED if the core part included ≥ 33.3% of both MIT and HIT. For all load calculations, the actual time spent in each of the three zones was considered, and training load (training impulse = TRIMP) was calculated as the product of duration (in minutes) and an intensity factor (1–3), as previously described (Foster et al. 2001; Solli et al. 2019).

To describe day‐by‐day variations in training characteristics across all athletes, training parameters were expressed relative to individual training volume (i.e., as % of daily mean) or frequency (e.g., as % of race days). Accordingly, daily training volume and load were categorized as very low (0%–50% of individual mean daily volume), low (50%–100%), moderate (100%–150%), and high (> 150%).

To analyze longitudinal changes in training load, the uncoupled acute‐to‐chronic work ratio (ACWR) was calculated for each day by dividing the 7‐day acute load by the chronic 7‐day mean load over the preceding 28 days. To account for differences in training routines throughout the season, the training year was separated into the general preparation period (May–October) and the competition period (November–April).

All recorded training sessions were checked for correct registration, and distribution and intensity classifications were verified via a semi‐structured interview with each participant during the data analysis phase of the study.

### 2.4 Statistical Analyses

All data are presented as mean ± standard deviation (SD). The normality of the data was tested by the visual inspection of histograms and the Shapiro–Wilk test (*α* = 0.05). Statistical comparisons to the mean were assessed using a one sample *t*‐test. The significance level (*p*‐value) was set to < 0.05. Effect sizes (ES) were calculated as Cohen's *d*, with the following interpretation criteria: 0.0–0.2, trivial; 0.2–0.6, small; 0.6–1.2, moderate; 1.2–2.0, large; and > 2.0, very large (Hopkins et al. 2009).

## 3 Results

### 3.1 Organization of Daily Training Load and Volume

**FIGURE 1:**
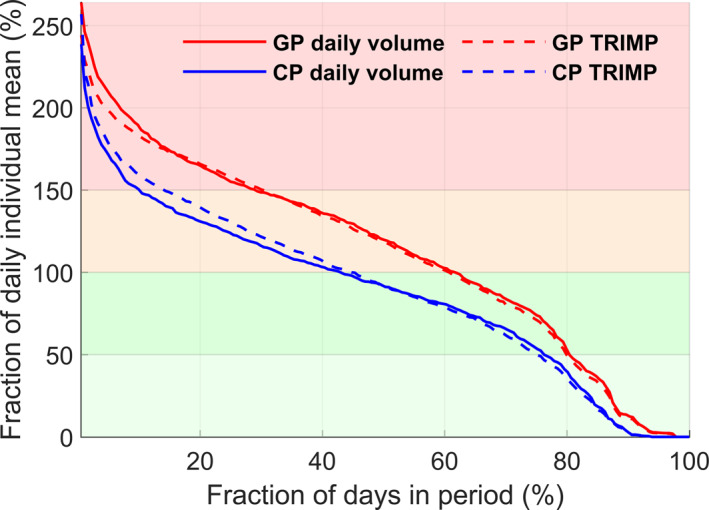
Distribution of daily training volume and load (training impulse; TRIMP) expressed as the percentage of individual daily mean during the general preparation period (GP) and competition period (CP) in 17 world‐class cross‐country skiers during a successful season. Graphs represent the group mean.

In total, 13,291 h and 8710 sessions of endurance training were analyzed (see Table 1 for annual training volumes). On average, the daily training volume and TRIMP were 128 ± 10 min and 145 ± 11 arbitrary units (AU), respectively. The distribution of daily training volume was 20 ± 3% very low, 28 ± 5% low, 32 ± 4% medium, and 19 ± 3% high‐volume days. A detailed distribution, along with the distribution of training load (TRIMP) during GP and CP, is presented in Figure 1. The mean weekly training volume was 996 ± 92 min during GP, compared to 769 ± 80 min during CP. Rest days occurred every 9.2 ± 4.4 days during GP (22.6 ± 9.0 days in total) and every 7.9 ± 2.9 days during CP (25.8 ± 7.3 days in total) on average. During both periods, the most frequently applied time gaps between rest days were back‐to‐back (consecutive) days (GP: 23.4 ± 15.9%, CP: 29.4 ± 23.3%) and ≥ 14 days (GP: 20.4 ± 18.5%, CP: 16.4 ± 13.2%).

**TABLE 1:** Annual training volume (mean ± SD [range]) and aerobic power during the analyzed season of 17 world‐class cross‐country skiers during a successful season.

Training forms	
Total training (h)	861.5 ± 73.7 (767.8–1022.2)
Endurance (h)	781.8 ± 62.0 (713.7–923.1)
Strength (h)	55.3 ± 22.3 (29.8–89.7)
Speed (h)	18.5 ± 7.7 (5.8–35.7)
Other (h)	5.5 ± 5.0 (0.0–14.4)
Intensity distribution	
LIT (h)	715.7 ± 59.0 (638.0–857.7)
MIT (h)	33.7 ± 8.6 (16.1–38.0)
HIT (h)	32.5 ± 6.7 (24.7–45.7)
Aerobic power	
VO_2max_ (mL·kg^−1^ min^−1^) men	81.6 ± 3.8^a^ (78.6–86.4)
VO_2max_ (mL·kg^−1^ min^−1^) women	71.3 ± 4.4 (67.4–80.0)

Abbreviations: HIT, high intensity training; LIT, low intensity training; MIT, moderate intensity training; VO_2max_, maximal oxygen uptake.

^a^ Mean values of 6 skiers due to missing test data in 4 skiers.

### 3.2 Organization of Volume and Intensity

**FIGURE 2:**
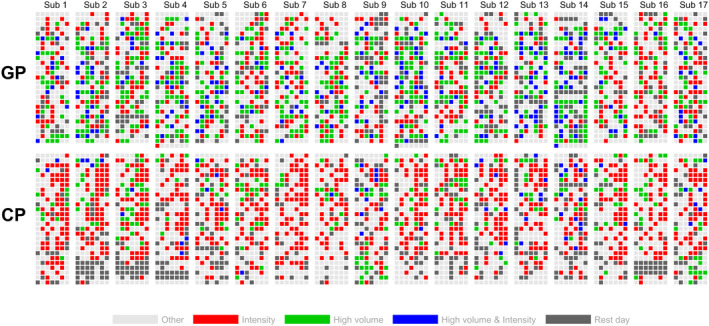
Individual distribution of the daily endurance training content in 17 world‐class cross‐country skiers during a successful season. High volume days (> 150% of individual daily mean, green), elevated intensity days (days on which moderate‐ to high‐intensity was applied, red), combined days (containing both high volume and intensity, blue), and rest days (dark gray) are shown separately for each skier, with each day in the general preparation period (GP, upper half) and competition period (CP, lower half) represented by a single square. First day in each period is represented by the upper left square and last day by the lower right square, with each row representing 1 week.

The individual application of days with high training volume, elevated intensity, a combination of both, and rest days throughout the year is shown in Figure 2. The mean numbers of days with high training volume during GP and CP were 36.7 ± 6.4 and 14.2 ± 6.2 days, respectively. The number of days with elevated intensity (containing MIT and HIT) was 50.0 ± 3.8 during GP and 60.4 ± 6.4 during CP. On average, the athletes combined high volume and intensity on 15.8 ± 7.5 days during GP and 3.8 ± 3.9 days during CP.

**FIGURE 3:**
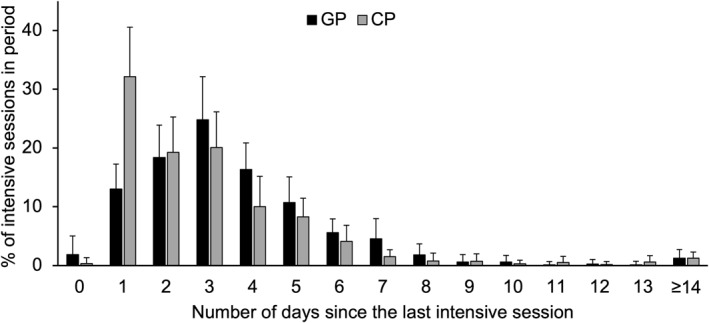
The distribution of different time intervals between intensive sessions (moderate‐ to high‐intensity) during the general preparation period (GP, black) and competition period (CP, gray) in world‐class cross‐country skiers during a successful season. Values represent the proportion of sessions for which each the respective time interval was used.

Intensive sessions were separated by 3.6 ± 0.3 days during GP and 3.0 ± 0.3 days during CP. The distribution of time intervals between intensive sessions during GP and CP is shown in Figure 3. Time intervals between sessions of the same intensity (MIT, MIXED, and HIT) were 8.4 ± 2.2, 24.0 ± 13.3, and 15.3 ± 8.9 days, respectively, during GP. The corresponding values for CP were 13.0 ± 10.6, 28.6 ± 24.9, and 22.1 ± 11.1 days, respectively.

During GP, the training volume was elevated both on the day before and the day after intensive sessions compared to the mean daily volume (before: 127 ± 13%, *p* < 0.001, *d* = 2.1; after: 116 ± 8%, *p* < 0.001, *d* = 2.0). During CP, the volume was reduced both before and after intensive sessions (before: 99 ± 7%, *p* < 0.001, *d* = 0.2; after: 87 ± 9%, *p* < 0.001, *d* = 1.4). Detailed information on endurance training loads before and after intensive sessions of different intensities, as well as competitions, is presented in Table 2.

**TABLE 2:** Endurance training load (training impulse; TRIMP) expressed as the percentage of daily mean prior to and after days with intensive sessions in world‐class cross‐country skiers during a successful season.

Type of intensive sessions	Endurance training load (TRIMP, expressed as % of mean daily TRIMP)			
	General preparation period		Competition period	
	Day before	Day after	Day before	Day after
MIT sessions	121 ± 15^c^	110 ± 14^b^	93 ± 16	101 ± 25
MIXED sessions	115 ± 28^a^	103 ± 34	91 ± 20	104 ± 24
HIT sessions	117 ± 17^b^	106 ± 16	98 ± 14	92 ± 23
Races	116 ± 29^a^	102 ± 20	101 ± 12	78 ± 11^d^

Abbreviations: HIT, high intensity training; MIT, moderate intensity training; MIXED, intensive sessions with ≥ 33.3% of moderate and high intensity.

^a^ Significantly different from the mean with small effect.

^b^ Significantly different from the mean with moderate effect.

^c^ Significantly different from the mean with large effect.

^d^ Significantly different from the mean with very large effect.

**FIGURE 4:**
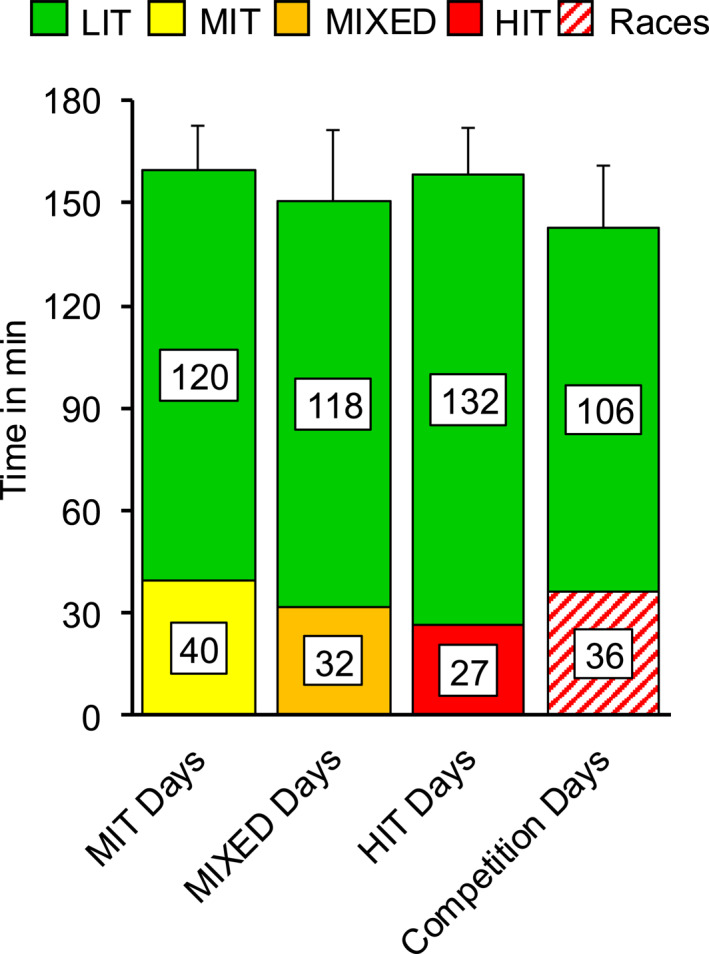
Mean duration of the intensive sessions' core part across different intensities (bottom part) and volume of low‐intensity training during the day that contained the respective type of intensive sessions (upper part) in 17 world‐class cross‐country skiers during a successful season. HIT, high intensity training; LIT, low‐intensity training; MIT, moderate intensity training; MIXED, intensive sessions with ≥ 33.3% of moderate‐ and high‐intensity.

**FIGURE 5:**
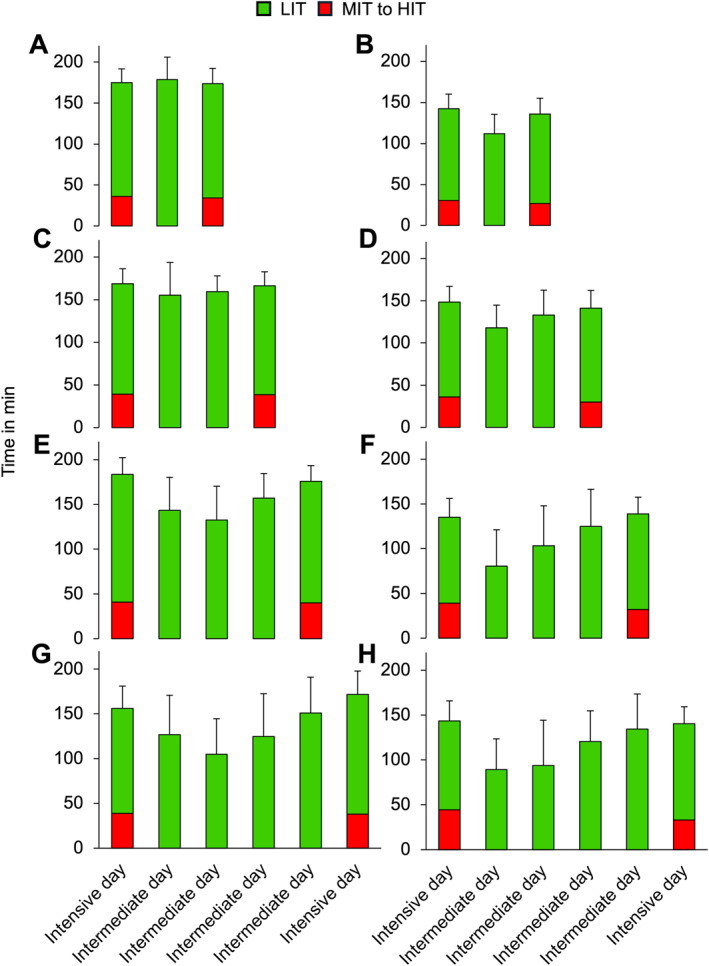
Mean daily endurance training volume on consecutive days with intensive sessions and intermediate days during the general preparation period (left) and competition period (right) across one (A and B), two (C and D), three (E and F), and four intermediate days (G and H) in 17 world‐class cross‐country skiers during a successful season. HIT, high‐intensity training; LIT, low‐intensity training; MIT, moderate‐intensity training.

The mean volume of LIT (including warm‐up and cool‐down) on days when intensive sessions were performed was 117 ± 12 min, whereas the total training time on those days was 152 ± 12 min (119 ± 9% of mean). Detailed analyses by the type of intensive session are presented in Figure 4. Figure 5 presents the mean daily volume on days including intensive sessions and intermediate days separated for different time intervals between intensive sessions. The presented scenarios make up for ∼63% of all intensive sessions.

### 3.3 Longitudinal Load Management

**FIGURE 6:**
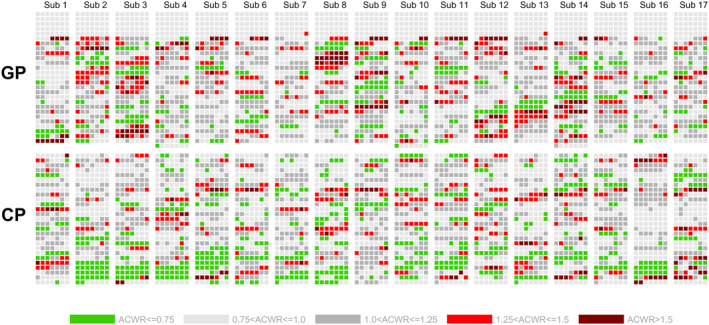
Individual distribution of the daily acute to chronic workload ratio (ACWR) in 17 world‐class cross‐country skiers during a successful season. Daily ACWR values are shown separately for each skier using squares colored from light green (low ACWR) to dark red (very high ACWR), with each day in the general preparation period (GP, upper half) and competition period (CP, lower half) represented by a single square. Initial 35 days are redundant due to the calculation of ACWR.

The longitudinal changes in training loads, expressed as ACWR, in the percentage of days with ratios < 0.75, 0.75–1.0, 1.0–1.25, 1.25–1.5, and > 1.5 were 13%, 32%, 36%, 13%, and 5% during GP and 19%, 40%, 30%, 9%, and 3% during CP. The individual daily distribution of ACWR across the entire season is shown in Figure 6.

## 4 Discussion

This is the first study to quantify day‐to‐day training characteristics of male and female world‐class XC skiers during their most successful season. The main findings were as follows:

1. The mean daily endurance training volume and load (TRIMP) were 128 ± 10 min and 145 ± 11 AU, respectively.
2. Training content was distributed in an alternating manner, combining days of high volume, days with elevated intensity, and rest days, with only 5% of days combining high volume and elevated intensity.
3. Intensive sessions were performed at 3.6‐day (GP) and 3.0‐day (CP) intervals, whereas sessions of the same intensity were separated by > 8 days.
4. A steady longitudinal training load strategy was applied, with only 18% of the days during GP and 12% during CP conducted with an ACWR ≥ 1.25.

### 4.1 Organization of Daily Training Load

The organization of daily endurance training load in the investigated athletes showed distinctly different patterns during GP and CP. During GP, approximately 30% of the days were performed with a high load, a fraction that was more than halved during CP, whereas the proportion of medium‐load days remained relatively similar across periods. In contrast, the proportion of very low‐ to low‐load days was ∼15% smaller during GP compared to CP. These novel findings reveal that world‐class XC skiers achieve higher training loads during GP by increasing the amplitude of daily training load on certain days (i.e., more days with high load) and by having fewer days with reduced load. This strategy of disseminating load and avoiding days with severe augmentations may contribute to a sustainable training process and help prevent unexpected underperformance (Talsnes et al. 2023). The load management observed in these skiers was supported by a recovery and unloading strategy that included interspersed rest days, most often applied as back‐to‐back rest days. The ∼48 annual rest days were complemented by ∼12 days (GP) and ∼18 days (CP) of very low load. This demonstrates a common pattern of regularly interspersed days of reduced training load combined with full rest days every 8–9 days. Although the role of days with very low load is less researched, passive rest days, however, have been proposed as essential for managing fatigue and enhancing recovery (Meeusen et al. 2013).

### 4.2 Organization of Volume and Intensity

Although the training load followed a relatively even distribution across days during GP, as described above, the underlying training content varied significantly. Days were typically dedicated to either high volume or intensive sessions and only rarely combined both (∼20 days). This concept may be reasonable for several reasons. First, it reflects a strategy of evenly distributing load by separating certain training components that, if performed together, would result in high physiological stress. This approach may be advantageous for the mentioned avoidance of unexpected underperformance (Talsnes et al. 2023). Second, performing high training volume on days with intensive sessions could compromise the quality of those sessions, which is crucial for maximizing simultaneous improvements in physiological, technical, and tactical aspects (S. B. Sandbakk et al. 2023). Although such alternations have already been proposed during the 1960s by Bill Bowerman's “hard‐easy principle,” it is noteworthy that these findings contrast with recent investigations of a world‐class cyclist, who regularly incorporated high‐intensity efforts within high‐volume sessions (Gallo et al. 2024). Whether these differences are due to sport‐specific demands or represent divergent training philosophies remains speculative. Future research should explore the effects of combining volume and intensity within the same day to better understand these discrepancies.

In addition to the separation of volume and intensity, the observed time gaps between days with intensive sessions provide novel insights into day‐to‐day periodization in world‐class skiers. The separation of 3–4 days during GP reflects the even distribution of approximately 10 monthly intensive sessions (Solli et al. 2017; Walther et al. 2024). In contrast, the shorter time gaps during CP are likely linked to back‐to‐back races and race reconnaissance, which typically includes intensity efforts performed the day before a race. Notably, during GP, training loads on the days before (and after) intensive sessions were moderate, regardless of the type of intensive sessions. Similarly, training volume on the days of intensive sessions was also moderate, reinforcing the above‐mentioned strategy of maintaining relatively consistent loads. Also, a significant reduction in load both before, during, and after intensive sessions would conflict with the overall annual training volume achieved by the athletes. Instead, the athletes in this study showed a tendency to favor reduced training volume on the days after intensive sessions, which became particularly apparent during CP and for longer time gaps between intensive sessions. This indicates that world‐class skiers can achieve high readiness to train and high training quality without substantially reducing training volume prior to intensive sessions while subsequently reduced strain might promote adaptations. Indeed, together with the mentioned race reconnaissance, this may also explain why training load was not substantially reduced on the day before races. Furthermore, these findings also illustrate that existing tapering theories with reduced pre‐competition load are not applicable throughout an entire season but rather limited to main competitions (Tønnessen et al. 2014; Mujika and Padilla 2003).

### 4.3 Longitudinal Load Management

The longitudinal load management of the athletes during the investigated season followed a traditional periodization approach, as previously described (Walther et al. 2023). When examining this strategy in more detail on a daily basis, the largest proportions of days fell within an ACWR range of 0.75–1.0 and 1.0–1.25, collectively accounting for two‐thirds of the days during both GP and CP. This reflects a primarily consistent longitudinal load strategy which, despite conceptual limitations of the ACWR, has been proposed to reduce injury risks (Bourdon et al. 2017). However, daily load variations in world‐class endurance athletes are sparsely described in the literature and it is reasonable to attribute the constant workload to a generally high training volume of the included athletes. Such high volumes neither allow for discontinuity nor for large spikes in training load, as these would either interrupt the training process or could contribute to unexpected underperformance, respectively (Talsnes et al. 2023; Mujika and Padilla 2000).

### 4.4 Strengths and Limitations

Although our findings provide invaluable lessons learned from the simultaneous investigation of 17 medal winning athletes, care should be taken when interpreting the present data. Including both men and women enhances the applicability of the findings, but possible sex differences in the micro periodization have not been specifically examined here. Also, the examination of athletes born in three different decades limits the influence time‐specific training philosophy. Still, executed training might have been influenced by the coaching and training philosophies in Norway. Furthermore, the application of TRIMP does not consider the use of different exercise modalities, which has been proposed to influence training load management (Ø. Sandbakk et al. 2021). Finally, the study does not consider the possible influence on race calendars and traveling routines for six athletes who had their best senior season during the COVID‐19 pandemic.

## 5 Conclusion

This study presents novel insights into the day‐to‐day periodization among world‐class cross‐country skiers. The key load management strategies identified here involve a system where the applied training sequences are distributed in an alternating manner (e.g., volume days vs. intensive days separated on different days), with a relatively stable acute to chronic load evolvement during training periods (∼15% of the days having an ACWR ≥ 1.25), regularly interspersed with days of reduced training load or rest every 8–9 days. On average, sessions with elevated intensity are separated by three to 4 days, usually evenly distributed during GP but more concentrated around competitions during CP. These findings not only bridge the gap between existing research on world‐class skiers where previous research either described broader, annual, or monthly training characteristics or focused more in detail on sessions designs but also provide useful guidelines for practitioners when planning a sustainable daily training organization. Finally, this study provides valuable points of departure for future research on the application of different day‐to‐day periodization strategies.

## Author Contributions

**Jacob Walther:** conceptualizing, formal analysis, investigation, writing – original draft, writing – review and editing. **Jan Kocbach:** formal analysis, investigation, writing – review and editing. **Øyvind Sandbakk:** conceptualizing, writing – original draft, writing – review and editing.

## Ethics Statement

The regional committee for medical and health research ethics waived the requirement for ethical approval for studies of this type. The ethics of the project adhered to institutional requirements at the Department of Neuromedicine and Movement Science, Norwegian University of Science and Technology, Norway. Approval for data security and handling was obtained from the Norwegian Center for Research Data (reference no. 419807).

## Consent

All participants provided written informed consent to participate in this study.

## Conflicts of Interest

The authors declare no conflicts of interest.

## Permission to Reproduce Material From Other Sources

The authors have nothing to report.

## Data Availability

Data are available on request from the corresponding author.
